# An unusual presentation of precursor T cell lymphoblastic leukemia/lymphoma with cholestatic jaundice: case report

**DOI:** 10.1186/1756-8722-2-12

**Published:** 2009-03-12

**Authors:** Kevin J Patel, Sahibzada U Latif, Wanderley M de Calaca

**Affiliations:** 1 Department of Medicine, Michigan State University, East Lansing, Michigan, USA; 2 Department of Pathology, EW Sparrow Hospital, Lansing, Michigan, USA

## Abstract

**Background:**

Cholestatic jaundice as a presenting symptom of Precursor T-lymphoblastic leukemia (T-ALL)/lymphoma (T-LBL) has never been reported in literature. Similarly, precursor T-ALL/T-LBL is characteristically negative for synaptophysin. We report the first case of a patient with precursor T-ALL/T-LBL who presented with cholestatic jaundice and aberrant tumor expression of synaptophysin.

**Case report:**

42 year old male presented with anorexia, nausea, jaundice, pale stools, dark urine and about 35 pound weight loss over the previous 3 weeks. The initial laboratory work was suggestive of cholestatic jaundice. Markedly elevated LDH (2025 U/L) and CA 19-9 (1778 u/ML) were also noticed. The CT scan of abdomen showed massive hepatomegaly with coarse echotexture with contracted gall bladder and normal sized common bile duct. Chest x-ray revealed a mediastinal mass with mediastinal widening. CT scan of the chest showed anterior mediastinal mass (16 cm × 10 cm). CT guided biopsy of the mass showed malignant lymphoma with diffuse proliferation of medium sized lymphoid cells. The neoplastic cells were positive for CD1a, CD3, CD4, CD5, CD8 and CD43 with aberrant expression of synaptophysin. PET CT scan again showed a large anterior mediastinal mass with diffuse liver involvement and abnormal activity in axial bones. CT guided liver biopsy and bone marrow biopsy revealed the same morphology and immunohistochemistry. Bone marrow aspirate showed 85% lymphoblasts. Thus, the diagnosis of precursor T-ALL/T-LBL was made and jaundice with elevated CA 19-9 were attributed to intrahepatic cholestasis.

**Conclusion:**

Our case illustrates an unusual presentation of hematological malignancies as cholestatic jaundice. It also indicates the non-specific nature of CA 19-9 for pancreaticobiliary malignancies. It is the first case report of neoplastic precursor T cell lymphoblasts with unusual expression of synaptophysin. Tissue biopsy with thorough immunohistochemistry is required to differentiate precursor T-ALL/T-LBL from thymoma and small cell carcinoma.

## Background

Precursor T-lymphoblastic leukemia (T-ALL)/lymphoma (T-LBL) is a neoplasm of lymphoblasts committed to the T-cell lineage. Clinically, if there are >25 percent bone marrow blasts with or without a mass lesion, the term precursor T-ALL is used. The term precursor T-LBL is used, if there is a mass lesion with less than 25 percent bone marrow involvement [1]. However, due to their biologic unity and significant clinical overlap, T-ALL and T-LBL are considered the same disease with different clinical presentations [2]. Our case highlights two unusual manifestations of precursor T-ALL/T-LBL, namely, the rare initial presentation of cholestatic jaundice and the aberrant expression of synaptophysin by the tumor cells both of which, to the best of our knowledge, have not been reported before.

## Case report

**Figure 1:**
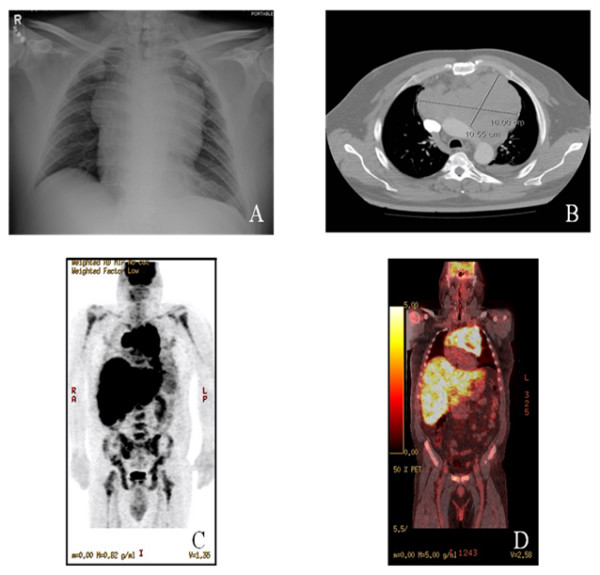
**Chest X-ray, CT chest and PET/CT images.** A. A chest x-ray (Frontal view) showing a huge mediastinal mass with evidence of mediastinal widening. B. CT scan of the chest showing 3 anterior mediastinal masses with the largest one being 16 cm × 10 cm. C. PET image revealing a very large anterior mediastinal mass and a diffuse liver involvement along with abnormal activity in both humeri, femura, and pelvic bones. D. Fusion PET/CT image showing a very large anterior mediastinal mass extending across the mediastinum from left to right and superiorly into the supraclavicular area. A diffuse involvement of the liver along with abnormal activity in both humeri, femura, and pelvic bones were also noticed.

42 year old obese (BMI-38) caucasian male presented to his primary care provider with complaints of insidious onset of anorexia, nausea, jaundice, pale stools, dark urine and about 35 pound weight loss over the previous 3 weeks. Outpatient laboratory work up revealed normal complete blood counts and basic panel but deranged liver function tests suggestive of cholestatic jaundice. Patient was then referred to a regional medical center for imaging studies. Ultrasound of abdomen showed multiple hyperechoic and hypoechoic liver lesions all less than 1 cm in size. The CT scan of abdomen showed massive hepatomegaly with coarse echotexture with contracted gall bladder and normal sized common bile duct. A chest x-ray (Figure 1A) obtained at the same time revealed a huge mediastinal mass with evidence of mediastinal widening. CT scan of the chest (Figure 1B) showed 3 anterior mediastinal masses with the largest one being 16 cm × 10 cm. In view of these imaging studies, the patient was referred to a tertiary care center for further work up. Upon arrival, we found the patient to be significantly icteric with some abdominal distension. There was smooth, non-tender liver edge palpable 10 cm below the right costal margin. No peripheral lymphadenopathy was noticed. The laboratory work on admission showed normal CBC and basic panel but persistently deranged liver function tests (Table 1).

**Table 1:** showing results of laboratory workup on admission at the tertiary care center (Day 0), during hospitalization (Day 7) and on outpatient follow up (day 100).

**Laboratory ** **Tests**	**Normal ** **Range**	**On admission** **(Day 0)**	**On Day 7 ** **(1 day before** **chemotherapy)**	**On Day ** **(after 3 cy of CD** **100 and 3 cy of** **Hyper CVAD ** **chemotherapy)**
ALT	2–45 U/L	130	270	33

AST	10–40 U/L	107	253	22

Alk. Phos.	0–120 U/L	894	1371	94

Total Protein	6.0–8.0 g/dL	6.7	5.9	6.2

Albumin	3.6–5.0 g/dL	3.3	3.0	3.3

Total Bili.	0.2–1.2 mg/dL	7.9	14.8	0.6

Direct Bili.	0.0–0.3 mg/dL	6.1	-	-

Indirect Bili.	0.0–0.9 mg/dL	1.8	-	-

PT	9–11.5 sec	12.5	-	10.6

LDH	100–225 U/L	2026	1838	220

Ammonia	13–37 μmol/L	72	-	-

CA 19-9	0–35 U/mL	1778.6	-	-

AST (Aspartate Transaminase), ALT (Alanine Transaminase), Alk. Phos. (Alkaline Phosphatase), Bili. (Bilirubin), PT (Prothrombin Time), LDH (Lactate Dehydrogenase) cy (cycles) and CD (cisplatin - dexamethasone).

**Figure 2:**
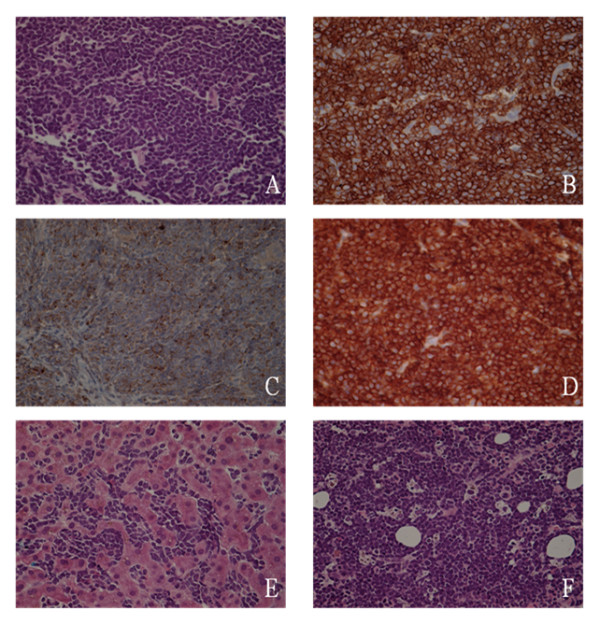
**H&E staining and immunohistochemical characteristics of tumor cells.** A. CT guided biopsy of the mediastinal mass (H&E staining) showing diffuse proliferation of medium sized lymphoid cells, exhibiting fine nuclear chromatin, inconspicuous nucleoli and scanty cytoplasm. Occasional mitosis with few scattered tangible body macrophages was noted. An area of necrosis was also noticed within the tumor. B. CD1a positive neoplastic cells from mediastinal biopsy aspirate. C. Synaptophysin positive neoplastic cells from mediastinal biopsy aspirate. D. CD3 positive neoplastic cells from mediastinal biopsy aspirate. E. H&E staining of the CT guided liver biopsy revealing the same tumor cell morphology as that of CT guided mediastinal mass biopsy. It also revealed intrahepatic cholestasis in the form of intracanalicular bile plugs. F. H&E staining of the bone marrow biopsy showing the same tumor cell morphology as that of CT guided mediastinal mass biopsy.

Additional work up was ordered which showed ceruloplasmin 5 mg/dl, AFP <1.3 ng/dl and serum ferritin 4686 ng/ml. ANA, anti-smooth muscle antibodies, HIV, along with EBV and CMV antibodies were all negative. CT guided biopsy of the mediastinal mass (Figure 2A) showed features of malignant lymphoma characterized by a diffuse proliferation of medium sized lymphoid cells, which exhibited fine nuclear chromatin, inconspicuous nucleoli and scanty cytoplasm. Occasional mitosis with few scattered tangible body macrophages was noted. The neoplastic cells were positive for CD1a (Figure 2B), CD3 (Figure 2D), CD4, CD5, CD8 and CD43. The neoplastic T cells also expressed synaptophysin (Figure 2C). However, the tumor was negative for CD20, kappa and lambda light chain immunoglobulins, CD34, TDT, cytokeratin AE1/AE3, cytokeratin 5.2 and CD56. The proliferation fraction (Ki-67) was 100%. Next, a PET CT scan (Figure 1C, D) was ordered which again showed a very large anterior mediastinal mass extending across the mediastinum from left to right and superiorly into the supraclavicular area. Diffuse liver involvement and abnormal activity in axial bones were also noticed. CT guided liver biopsy (Figure 2E) and bone marrow biopsy (Figure 2F) revealed the same tumor cell morphology with identical immunohistochemistry. A 200-cell count with differential performed on bone marrow aspirate showed 85% lymphoblasts, 2% small mature lymphocytes, 6% segmented neutrophils, 2% intermediate granulocytic precursors and 5% erythroid precursors. Ultrasound of bilateral testes was negative. MRI brain showed no masses or infarcts. Lumbar puncture was normal with no lymphoblasts. Thus, the diagnosis of precursor T-ALL/T-LBL was made. Since this patient had more than 25 percent T lymphoblasts in his bone marrow aspirate, his disorder could be best described as precursor T-ALL. After having considered the highly deranged liver function tests, per the recommendations of the treating oncologist, the treatment was initiated with cisplatin and dexamethasone (CD) for a total of 3 cycles to be followed by 4 cycles of Hyper CVAD. Follow up on day 100 after the completion of 3 cycles of Hyper CVAD revealed a normal hepatic panel and normal LDH (Table 1) with the patient scheduled for a fourth round of Hyper CVAD and a subsequent referral to a bone marrow transplant center.

## Discussion

Cholestatic jaundice as an initial presentation of acute lymphoblastic leukemia (ALL) is exceedingly rare [3,4]. A histopathological study of liver from autopsies of untreated patients diagnosed with leukemia or lymphoma did not reveal any evidence of cholestasis [5]. Majority of the cases reported describe cholestatic jaundice due to extrahepatic bile duct obstruction as a presenting feature of acute leukemia [6,7] Whereas Kelleher et al have reported intrahepatic cholestasis as initial presentation of precursor B-ALL in two children [8]; our literature search did not reveal any case of intrahepatic cholestasis in association with precursor T-ALL/T-LBL. We report the first case of a patient with precursor T-ALL/T-LBL who presented with intrahepatic cholestasis.

This patient had presented with painless jaundice, pale stools, direct hyperbilirubinemia, non-dilated common bile duct and multiple liver lesions suggestive of intrahepatic cholestasis. The significantly elevated serum CA 19-9 level was later attributed to the cholestatic jaundice rather than primary hepatobiliary and pancreatic malignancies [3]. This represents the nonspecific nature of this tumor marker [9].

This patient had a bulky anterior mediastinal mass. Masses in the anterior compartment are more likely to be malignant than those found in the other mediastinal compartments [10]. Thymomas are the most common anterior mediastinal primary neoplasms in adults [11]. Symptoms due to myasthenia gravis or other tumor-related syndromes are present in 35 percent of patients with thymoma at diagnosis [5]. Small cell lung cancer presents most commonly as a large hilar mass with bulky mediastinal adenopathy. Therefore, tissue biopsy with thorough immunohistochemical analysis is important to differentiate between thymomas, small cell lung cancers and lymphomas. Thymomas are composed of a mixture of neoplastic epithelial cells and non-neoplastic T lymphocytes, small cell carcinomas are characterized by small "blue" malignant cells about twice the size of lymphocytes, whereas T cell Lymphomas are composed of neoplastic T lymphocytes. The tumor cells in this patient were cytokeratin negative indicating their non-epithelial origin and thus, making the diagnosis of thymoma unlikely. These cells were also negative for most of the neural and neuroendocrine markers except for synaptophysin. This helped rule out small cell carcinoma. Further, these cells were positive for CD1a, sCD3, CD4/CD8 double positive indicating that neoplastic cells were in fact T cell lymphoblasts [2] more specifically, the common thyomocytes which represent an intermediate intrathymic maturation stage for T lymphoblasts. The lack of tumor cell expression of CD34 and TdT markers, as seen in this patient, is more common in precursor T-ALL than in precursor B-ALL [12]. Thus, tissue biopsy with thorough immunohistochemistry is required to differentiate T-ALL from thymoma and small cell carcinoma.

It was, however, unusual that these T cell lymphoblasts had aberrant expression of synaptophysin, an immunocytochemical marker for neuroendocrine differentiation (Figure 2C). Our literature search did not reveal synaptophysin positivity in any case of lymphoma or leukemia. Whether this aberrant expression of synaptophysin in precursor T-ALL/T-LBL carries any prognostic significance remains to be evaluated.

## Conclusion

To our knowledge, this is the first case report of precursor T-ALL/T-LBL presenting as cholestatic jaundice in an adult and also of neoplastic precursor T cell lymphoblasts expressing synaptophysin. It also indicates the non-specific nature of CA 19-9 for pancreaticobiliary malignancies. Tissue biopsy with thorough immunohistochemistry is required to differentiate precursor T-ALL/T-LBL from thymoma and small cell carcinoma.

## Consent

Written informed consent was obtained from the patient for publication of this case report and accompanying images. A copy of the written consent is available for review by the Editor-in-Chief of this journal.

## Competing interests

The authors declare that they have no competing interests.

## Authors' contributions

KP and SL assembled, analyzed and interpreted the patient data regarding the hematological disease. All authors contributed to writing the manuscript. All authors read and approved the final manuscript.
